# GIV is a promising novel poor prognostic factor in liver hepatocellular carcinoma

**DOI:** 10.1097/MD.0000000000029645

**Published:** 2022-08-12

**Authors:** Zhenzhen Zou, Yibin Sun, Lin Wang, Sai Ma, Chunrong Sun, Yu Zhou, Guorong Yang

**Affiliations:** a Department of Laboratory, Dushuhu Public Hospital Affiliated to Soochow University, Suzhou, Jiangsu Province, China; b Department of Gastroenterology, Suzhou Affiliated Hospital of Nanjing Medical University, Suzhou Municipal Hospital, Gusu School, Nanjing Medical University, Suzhou, Jiangsu Province, China; c Department of Central Laboratory, Suzhou Affiliated Hospital of Nanjing Medical University, Suzhou Municipal Hospital, Gusu School, Nanjing Medical University, Suzhou, Jiangsu Province, China; d Department of General Surgery, Suzhou Affiliated Hospital of Nanjing Medical University, Suzhou Municipal Hospital, Gusu School, Nanjing Medical University, Suzhou, Jiangsu Province, China; e Department of General Surgery, Second Affiliated Hospital of Soochow University, Souzhou Jiangsu, China.

**Keywords:** bioinformatics analysis, GIV, liver hepatocellular carcinoma, prognostic, TCGA

## Abstract

Numerous studies have implicated Gα-interacting, vesicle-associated protein (GIV) in the development and metastasis of various cancers. However, its role remains unclear in liver hepatocellular carcinoma (LIHC). We aimed to demonstrate the relationship between GIV and LIHC based on The Cancer Genome Atlas database. We use the Gene Expression Profiling Interactive Analysis and UALCAN to explore the expression of GIV and the survive analysis of GIV in patients with LIHC, genetic alteration analysis, immune infiltration analysis, functional enrichment, protein-protein interaction network analyses, and transcription factor targets of GIV-correlated genes and GIV-interacting genes were performed this study. GIV expression was significantly elevated in LIHC tissues. Remarkable correlation was established between GIV expression and LIHC pathological stage. Low expression of GIV in tumor tissues had a better prognosis than GIV-high expression. GIV alteration frequency was 1.44% in patients with LIHC. GIV-unaltered patients had better survival than GIV-altered ones. Moreover, GIV expression level in LIHC significantly correlated with the infiltration level of immune cells and cancer-associated fibroblasts. The functions of differentially expressed GIVs are associated with the cell cycle pathway. Our data imply that E2F4, E2F1, MYC, and MYCN are key transcription factors for GIV-correlated genes and GIV-interacted genes. GIV may be an adverse prognostic factor for patients with LIHC; it also can be a potential therapeutic target against LIHC. Further studies are required to validate our findings.

## 1. Introduction

Liver hepatocellular carcinoma (LIHC) is the third principal cause of cancer mortality with rapidly rising incidence worldwide.^[1,2]^ Even if the intrahepatic cancer tissue can be excised or ablated, the residual tumor microenvironment may lead to recurrence and disease progresses for most patients.^[3]^ Thus, identification of specific and sensitive LIHC biomarkers and potential therapeutic targets is critical for early diagnosis, treatment, and better prognosis.

Gα-interacting, vesicle-associated protein (GIV) has numerous cellular functions. It consists of 1870 amino acid residues and has a relative molecular weight of 220−250 × 10^3^. It is also known as Akt phosphorylation enhancer, Girdin (microfilament attachment protein), or hook-associated protein 1.^[4–7]^ As a member of the CCDC88 protein family, it is also named CCDC88A and can bind to and activate GA (GBA).^[8]^ Mounting evidence shows that GIV directly or indirectly regulates tumor immunity and biological phenotype, thereby modulating angiogenesis and tumorigenesis. GIV is implicated in tumorigenesis,^[9,10]^ but its role in LIHC remains unclear.

The development of second-generation sequencing technology and public databases like The Cancer Genome Atlas (TCGA) provides a better methodological platform for tumor diagnosis and treatment.^[11,12]^ With this aim in mind, in this article, we analyzed the tumor types of GIV using the TCGA database. By using bioinformatics analysis, we analyzed the coding genes of GIV and its main functional regions and discussed the expression pattern, potential function, and potential prognostic value of GIV in LIHC.

## 2. Materials and Methods

### 2.1. Gene expression analysis

We used TIMER2 (http://timer.cistrome.org/), a reliable and intuitive tool that allows comprehensive assessment of immune invasion across multiple kinds of cancers^[13]^ by inputting CCDC88A into the “Gene_DE” module and evaluating GIV expression in different tumors or tumors versus adjacent normal tissues in TCGA data resource. UALCAN data resource (http://ualcan.path.uab.edu/analysis.html) constitutes a comprehensive web tool that is interactive and user-friendly for the analysis of cancer OMICS data and allows easy access to freely accessible cancer OMICS data (TCGA, MET500, and CPTAC).^[14]^ We used the “Gene Analysis” module on UALCAN and the “LIHC” data set to obtain GIV expression data based on sample types. Using the “Pathological Staging Map” Gene Expression Profiling Interactive Analysis 2 (GEPIA2; http://gepia2.cancer-pku.cn/#index) module, which contains RNA sequence expression data for 9736 tumors along with 8587 nonmalignant tissue samples,^[15]^ a violin map of GIV expression was obtained at various LIHC pathological stages. The log2 (transcripts per million + 1) for log scale was applied for box and violin plot analyses. The Student *t* test was adopted to generate the *P* value, and *P* = 0.01 was set as the cutoff.

### 2.2. Survival prognosis analysis

We employed the “Survival Map” module on GEPIA2 to assess overall survival (OS), as well as disease-free survival correlation with GIV expression in LIHC of the TCGA database. We used cutoff-high (50%) and cutoff-low (50%) threshold values as the thresholds to separate the high expression group from the low expression group. The survival graph module of GEPIA2 was obtained through “Survival Analysis”. Prognosis was assessed with the Kaplan-Meier curve analysis. The *P* value cutoff was 0.05.

### 2.3. Analysis of genetic alteration

By signing in cBioPortal (https://www.cbioportal.org/), a comprehensive web tool for visualizing and analyzing multidimensional cancer genomics data,^[16]^ “TCGA Pan Cancer Atlas Studies” was selected in the “Quick Select” section and the term “CCDC88A” input to query the gene change characteristics of GIV. In the “Cancer Types Summary” module, we observed the change frequency and copy number alterations along with mutation type results of LIHC in the TCGA database. The “comparison” unit was used to obtain data on OS, disease-free survival, and PSF differences in cancer cases with or without GIV gene changes in the TCGA data set. The log-rank test was employed for Kaplan-Meier analysis, and *P* value cutoff was set at 0.01. The gene changes of GIV contain mutation, amplification, and deep deletion.

### 2.4. Immune infiltration analysis

Herein, relationship of GIV level with immune cell infiltration was evaluated using gene module on TIMER2.0. TIMER, EPIC, CIBERSORT, XCELL, CIBERSORT-ABS, MCPCOUNTER, and QUANTISEQ algorithms were used to assess immune invasion. *P* values along with partial correlation (Cor) values were determined using purity-adjusted Spearman rank correlation test and data visualized on heat maps and scatter plots.

### 2.5. Enrichment analysis in the GIV-related gene

STRING (https://string-db.org/) collects, scores, and integrates all freely accessible protein-protein interaction data and complements it with computational predictions of potential functions.^[17]^ First, we adopted the “Similar Gene Detection” module on GEPIA2 to assess the top 100 GIV-linked targeting genes on the basis of tumor versus normal tissue TCGA data sets. We used “CCDC88A” as query and “Homo sapiens” as organism on STRING to develop a protein-protein interaction network for GIV.

DAVID 6.8 (https://david.ncifcrf.gov/home.jsp) offers a comprehensive set of functional annotation tools for investigators to comprehend the biological meaning of large gene sets.^[18]^ Herein, gene ontology (GO) along with the Kyoto Encyclopedia of Genes and Genomes (KEGG) pathway analyses of GIV and closely linked neighbor genes obtained from GEPIA2 and STRING were abstracted from DAVID; a *P* value was set as the significance threshold. The GO enrichment analysis consisted of biological processes and cellular components along with molecular function.

TRRUST (https://www.grnpedia.org/trrust/) contains 8444 transcription factor (TF) target modulatory relationships on 800 human TFs to facilitate data-driven decisions.^[19]^ Here, the “Search” module in the TRRUST data resource was used to obtain information regarding the modulation of these cross talks. We then used the “correlation analysis” unit on GEPIA2 to carry out a pairwise Pearson correlation analysis between GIV and selected genes. Log2 transcripts per million was adopted to construct a dot plot, and the correlation coefficient (R) was indicated.

## 3. Results

### 3.1. Aberrant GIV expression in patients with LIHC

**Figure 1.**
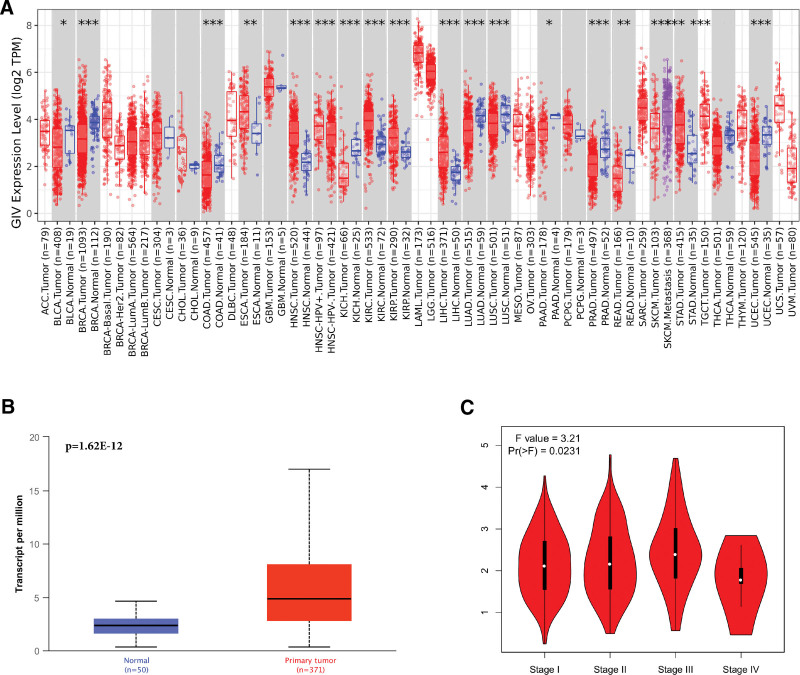
Expression level of the GIV gene in different tumors. (A) The expression status of GIV gene in different tumors (*P < 0.05, **P < 0.01, ***P < 0.001). (B) The gene expression level of GIV between normal tissue and LIHC tissue (*P* < 0.05). (C) The expression level of the GIV gene in the main pathological stages (stages I to IV) of LIHC. BLCA = bladder urothelial carcinoma, BRCA = breast invasive carcinoma, CESC = cervical squamous cell carcinoma and endocervical adenocarcinoma, CHOL = cholangiocarcinoma, COAD = colon adenocarcinoma, ESCA = esophageal carcinoma, GBM = glioblastomamultiform, GIV = Gα-interacting, vesicle-associated protein, HNSC = head and neck squamous cell carcinoma, KIRC = kidney renal clear cell carcinoma, KIRP = kidney renal papillary cell carcinoma, LIHC = liver hepatocellular carcinoma, LUAD = lung adenocarcinoma, LUSC = lung squamous cell carcinoma, PRAD = prostate adenocarcinoma, READ = rectal adenocarcinoma, SARC = sarcoma, STAD = stomach adenocarcinoma, THCA = thyroid carcinoma, TPM = transcripts per million, UCEC = uterine corpus endometrial carcinoma.

We evaluated GIV mRNA expression patterns in LIHC and normal tissues by using the TCGA database (Raw Data 1, Supplemental Digital Content, http://links.lww.com/MD/G960). GIV expression increased at mRNA level. Evaluation of GIV expression in various cancer types on TCGA revealed that its expression level was higher in contrast with that in the paired control tissues (Fig. 1A). The UALCAN data set exhibited higher GIV expression in LIHC cases (371) than in normal control tissues (50; Fig. 1B; *P* = 1.62 × 10^−12^). Assessment of correlation between GIV expression and LIHC pathological stage using the “Pathological Stage Plot” module on GEPIA2 revealed remarkable correlation between GIV expression with pathological stage (Fig. 1C; *P* = 0.023). LIHC progression correlated with increasing GIV expression. These data illustrate that GIV plays a remarkable role in LIHC tumorigenesis and progression.

### 3.2. Survival analysis of GIV in LIHC patients

**Figure 2.**
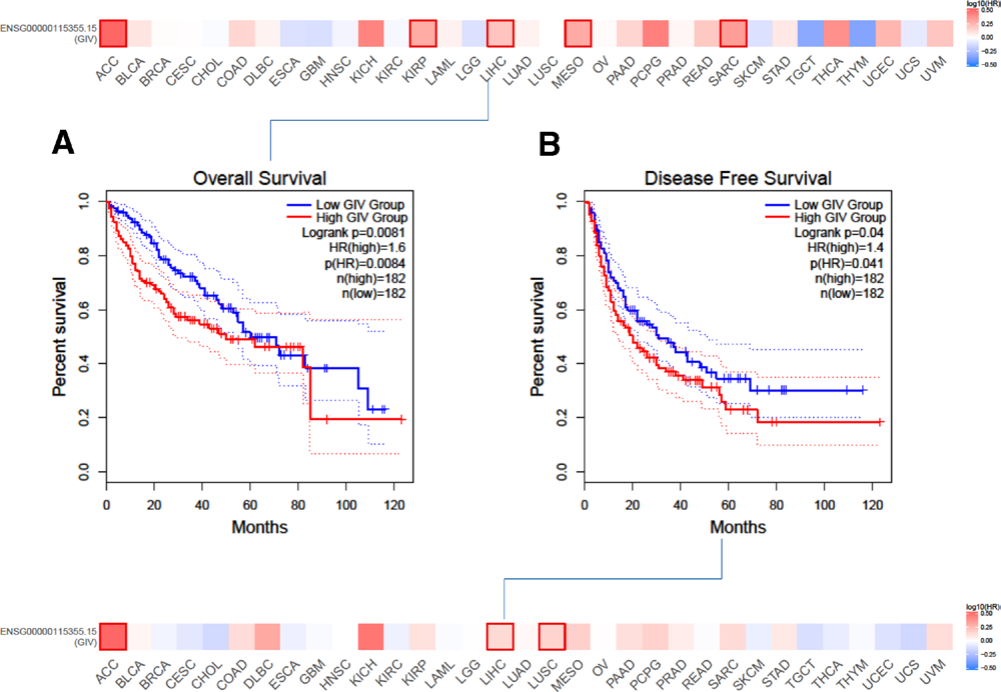
High expression of GIV is associated with poor OS and DFS in patients with LIHC. (A) The Kaplan-Meier curves of OS in LIHC. (B) The Kaplan-Meier curves of DFS in LIHC. BLCA = bladder urothelial carcinoma, BRCA = breast invasive carcinoma, CESC = cervical squamous cell carcinoma and endocervical adenocarcinoma, CHOL = cholangio carcinoma, COAD = colon adenocarcinoma, DFS = disease-free survival, ESCA = esophageal carcinoma, GBM = glioblastomamultiform, GIV = Gα-interacting, vesicle-associated protein, HNSC = head and neck squamous cell carcinoma, KIRC = kidney renal clear cell carcinoma, KIRP = kidney renal papillary cell carcinoma, LIHC = liver hepatocellular carcinoma, LUAD = lung adenocarcinoma, LUSC = lung squamous cell carcinoma, OS = over survival, PRAD = prostate adenocarcinoma, READ = rectal adenocarcinoma, SARC = sarcoma, STAD = stomach adenocarcinoma, THCA = thyroid carcinoma, UCEC = uterine corpus endometrial carcinoma.

Based on the expression level, we stratified cancer cases into high (182) and low (182) GIV expression groups. Analysis of correlation between GIV expression and LIHC patient prognosis in TCGA and GEO data sets using GEPIA2 revealed that high GIV expression correlates with dismal OS (*P* = 0.0084) and DFS (*P* = 0.041; Fig. 2A and B).

### 3.3. Genetic alteration analysis data of GIV in patients with LIHC

**Figure 3.**
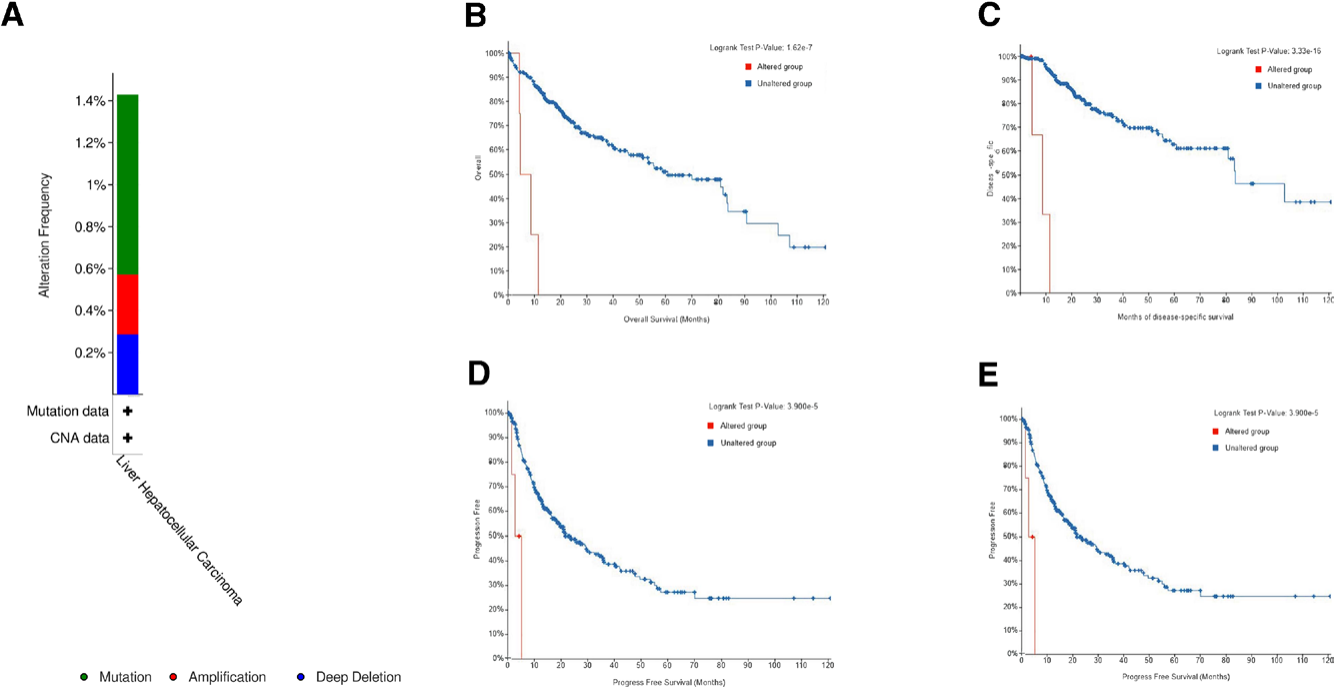
Mutation feature of GIV in LIHC. (A) The alteration frequency of GIV for LIHC. (B–E) The potential correlation between mutation status and overall, disease-specific, disease-free, and progression-free survival of LIHC. CNA = copy number alteration, GIV = Gα-interacting, vesicle-associated protein, LIHC = liver hepatocellular carcinoma.

Analysis of GIV genetic alteration in the TCGA LIHC cohort revealed an alteration frequency of 1.44% (Fig. 3A). The alteration type contains mutation (0.86%), amplification (0.29%), and deep deletion (0.29%). The protein change was the missense mutation of I212V and D393E. Next, we stratified the cancer cases into the altered and unaltered groups based on GIV alteration in patients with LIHC. Of the 343 LIHC cases, 5 GIV-altered cases had poor disease-specific survival (*P* = 3.33 × 10^−16^, *q* = 1.33 × 10^−15^). Of 303 LIHC cases, 4 GIV-altered cases had poor DFS (*P* = 2.44 × 10^−9^, *q* = 4.87 × 10^−9^). Of 352 LIHC cases, 5 GIV-altered cases had poor OS (*P* = 1.62 × 10^−7^, *q* = 2.16 × 10^−7^). Of 352 LIHC cases, 5 GIV-altered cases had poor PFS (*P* = 3.90 × 10^−5^, *q* = 3.90 × 10^−5^; Table 1). The Kaplan-Meier plotter approach was employed to present survival data (Fig. 3B–E).

**Table 1:** The enrichment analysis of different expressed Gα-interacting, vesicle-associated proteins and 100 most frequently altered neighboring genes in liver hepatocellular carcinoma.

Category	Term	Description	Count	FDR
GOTERM_BP	GO:0051301	Cell division	21	6.32 × 10^−12^
GOTERM_BP	GO:0007062	Sister chromatid cohesion	14	8.10 × 10^−12^
GOTERM_BP	GO:0007067	Mitotic nuclear division	14	3.98 × 10^−7^
GOTERM_CC	GO:0005654	Nucleoplasm	49	4.82 × 10^−12^
GOTERM_CC	GO:0005813	Centrosome	19	1.41 × 10^−9^
GOTERM_CC	GO:0000776	Kinetochore	11	1.41 × 10^−9^
GOTERM_CC	GO:0005819	Spindle	12	3.04 × 10^−9^
GOTERM_CC	GO:0000777	Condensed chromosome kinetochore	10	4.39 × 10^−8^
GOTERM_CC	GO:0005874	Microtubule	14	4.53 × 10^−7^
GOTERM_CC	GO:0030496	Midbody	10	9.02 × 10^−7^
GOTERM_CC	GO:0005634	Nucleus	54	3.31 × 10^−5^
GOTERM_CC	GO:0005829	Cytosol	34	0.00435
GOTERM_CC	GO:0005737	Cytoplasm	46	0.006187
GOTERM_MF	GO:0005515	Protein binding	82	1.22 × 10^−8^
GOTERM_MF	GO:0005524	ATP binding	28	4.04 × 10^−6^
GOTERM_MF	GO:0008017	Microtubule binding	11	1.77 × 10^−5^
GOTERM_MF	GO:0004672	Protein kinase activity	10	0.006101
KEGG_PATHWAY	hsa04110	Cell cycle	6	0.02109

### 3.4. Immune infiltration analysis of GIV in LIHC patients

**Figure 4.**
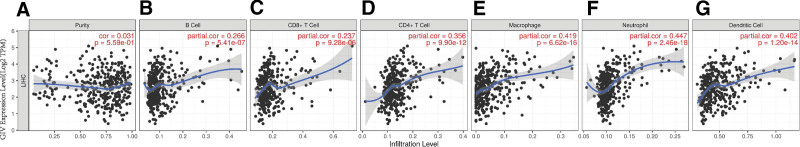
Correlation analysis between different expressed GIV and immune cell infiltration in liver hepatocellular carcinoma. The correlation between the expression of GIV and the abundance of (A) purity, (B) B cell, (C) CDS+ T cell, (D) CD4+ T cell, (E) macrophage, (F) neutrophil, and (G) dendritic cell. Cor = correlation, GIV = Gα-interacting, vesicle-associated protein, TPM = transcripts per million.

**Figure 5.**
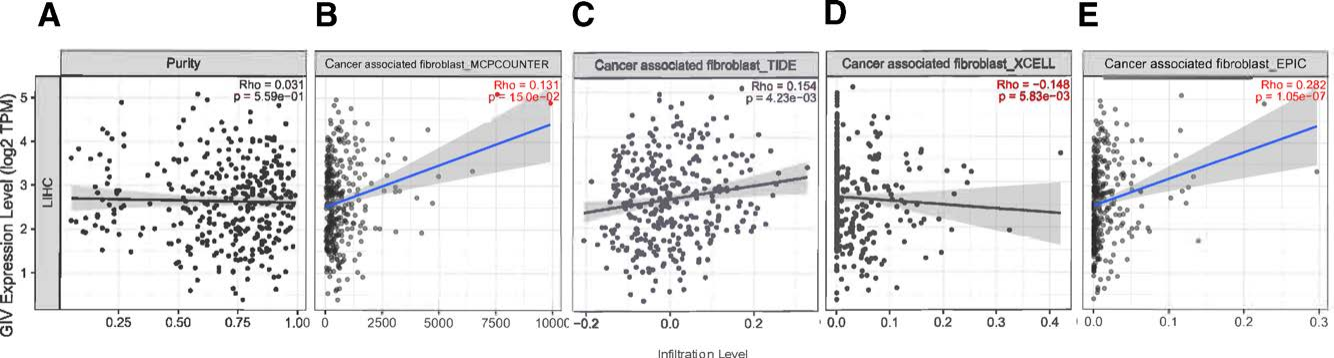
The expression level of GIV and the infiltration level of cancer-associated fibroblasts in liver hepatocellular carcinoma. The correlation analysis between GIV levels and cancer-associated fibroblast levels revealed though (A) purity, (B) EPIC, (C) MCPCOUNTER, (D) TIDE, and (E) XCELL. GIV = Gα-interacting, vesicle-associated protein, TPM = transcripts per million.

Tumorigenesis is strongly linked to the tumor microenvironment, and tumor-invading immune cells, which are components of the tumor microenvironment, also affect LIHC prognosis. Evaluation correlation between the invasion level of various immune cells and GIV gene expression using TIMER, XCELL, CIBERSORT, QUANTISEQ, EPIC, MCPCOUNTER, and CIBERSORT-ABS revealed positive correlation of GIV expression with invasion by B cells (Cor = 0.266, *P* = 5.41 × 10^−7^), CD8^+^ T cells (Cor = 0.237, *P* = 9.28 × 10^−6^), CD4^+^ T cells (Cor = 0.356, *P* = 9.90 × 10^−12^), macrophages (Cor = 0.419, *P* = 6.62 × 10^−16^), neutrophils (Cor = 0.447, *P* = 2.46 × 10^−18^), and dendritic cells (Cor = 0.402, *P* = 1.20 × 10^−14^; Fig. 4). Cancer-associated fibroblasts in the tumor microenvironment stroma influence the function of various tumor-invading immune cells. Moreover, positive correlation of GIV expression level in LIHC with the infiltration level of cancer-associated fibroblasts was revealed by EPIC (Cor = 0.282, *P* = 1.05 × 10^−7^), MCPCOUNTER (Cor = 0.131, *P* = 1.50 × 10^−2^), and TIDE (Cor = 0.154, *P* = 4.23 × 10^−3^) analyses. However, the XCELL analysis revealed negative correlation (Cor = −0.148, *P* = 5.83 × 10^−3^; Fig. 5).

### 3.5. Genetic coexpression, neighbor gene network, and enrichment analysis of GIV in LIHC patients

**Figure 6.**
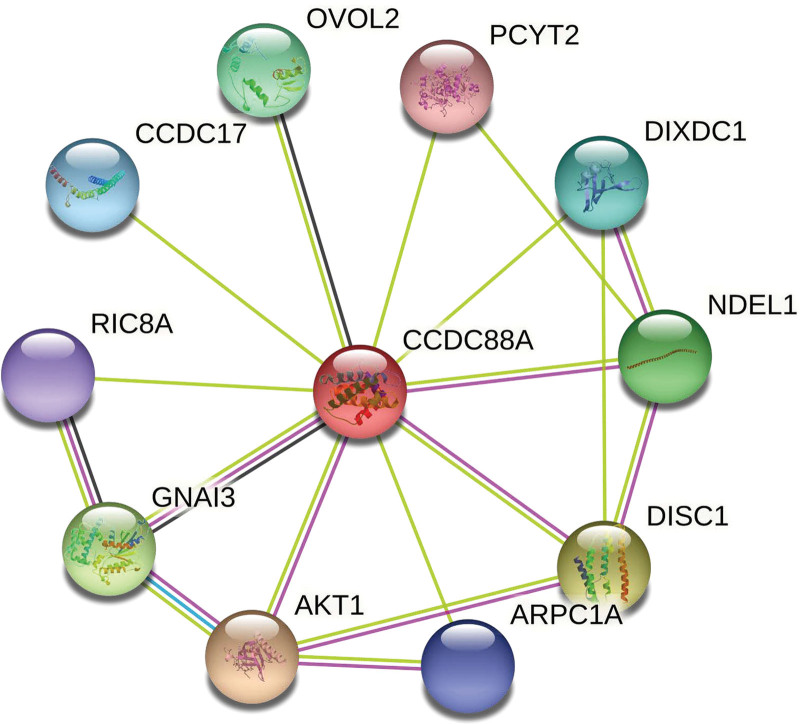
Genetic coexpression analysis of GIV. The available experimentally determined GIV-binding proteins. GIV = Gα-interacting, vesicle-associated protein.

To assess the role of GIV in tumorigenesis, we sought to identify GIV-interacting proteins and genes associated with GIV expression using the pathway enrichment analysis. The STRING analysis identified the top 10 related genes *AKT1*, *ARPC1A*, *CCDC17*, *DISC1*, *DIXDC1*, *GNAI3*, *NDEL1*, *OVOL2*, *PCYT2*, and *RIC8A* (Fig. 6) as GIV-interacting proteins. The GEPIA2 analysis TCGA LIHC expression data identify the top 100 genes associated with GIV expression as *KIF20B*, *SGOL2*, *SENP1*, *NUP107*, *HNRNPR*, *DBF4*, *KIF18A*, *CENPE*, *RACGAP1*, *INCENP*, *FAM208A*, *PPP1CC*, *ZWILCH*, *CKAP2*, *TUBA1B*, *SPDL1*, *R3HDM1*, *MCM8*, *C4orf46*, *CKAP2L*, *BUB1*, *SASS6*, *POLQ*, *APAF1*, *MSH2*, *UBA3*, *DR1*, *FBXO5*, *TARDBP*, *TAF1B*, *RQCD1*, *PPHLN1*, *ARHGAP11A*, *UBE2E1*, *KIF11*, *SCLT1*, *MTBP*, *NEDD1*, *ERCC6L*, *TPX2*, *C2orf44*, *HNRNPA2B1*, *RPAP3*, *KIF23*, *RAD51AP1*, *TOPBP1*, *HNRNPLL*, *SGOL1*, *CDK2*, *TMEM237*, *CBX1*, *BRPF1*, *IKBIP*, *DCLRE1B*, *UBE2N*, *KIF15*, *MGME1*, *GSG2*, *DENR*, *LMNB1*, *DLGAP5*, *ANLN*, *C3orf38*, *CDC7*, *MKRN2*, *NEMP1*, *SUV39H2*, *SPAST*, *MELK*, *PRR11*, *BUB1B*, *HAT1*, *COMMD2*, *ARL8B*, *CKAP5*, *VHL*, *UBE2E3*, *KIF18B*, *WASF1*, *SAP130*, *GTF2H3*, *KIAA1524*, *PPP1R8*, *TTK*, *CENPA*, *VRK2*, *MCM10*, *WHSC1*, *CTD-2510F5.4*, *FOXM1*, *TICRR*, *DDIAS*, *GTF2H1*, *CDCA8*, *ZNF639*, *TRA2B*, *SMC4*, *CPSF6*, *GINS1*, and *NRAS*. We then combined these two data and performed KEGG along with GO analyses. The KEGG data illustrated that “cell cycle” might contribute to impact of GIV on tumor onset (Table 1). The GO data illustrated that in the BP category, cell division and mitotic nuclear division coupled with sister chromatid cohesion were linked to tumorigenesis and progress of LIHC. Nucleoplasm, centrosome, kinetochore, spindle, condensed chromosome kinetochore, microtubule, midbody, nucleus, cytosol, and cytoplasm were highly enriched in the cellular component category. In the molecular function category, protein binding, ATP binding, microtubule binding, and protein kinase activity were enriched (Table 2).

**Table 2:** Key regulated factor of Gα-interacting, vesicle-associated protein and correlated genes in liver hepatocellular carcinoma.

Key TF	Description	Regulated genes	*P* value	FDR
E2F4	E2F TF 4, p107/p130 binding	*CDK2*, *MCM10*, *TOPBP1*, and *TTK*	4.36 × 10^−6^	2.62 × 10^−5^
E2F1	E2F TF 1	*APAF1*, *DBF4*, *FOXM1*, *MCM8*, and *RACGAP1*	0.000502	0.00151
MYC	v-myc myelocytomatosis viral oncogene homolog (avian)	*CDK2*, *FOXM1*, and *HNRNPA2B1*	0.0129	0.0258
MYCN	v-myc myelocytomatosis viral related oncogene, neuroblastoma derived (avian)	*MCM10* and *MCM8*	0.0203	0.0305

### 3.6. TF targets of GIV-correlated genes and GIV-interacting genes in patients with LIHC

**Figure 7.**
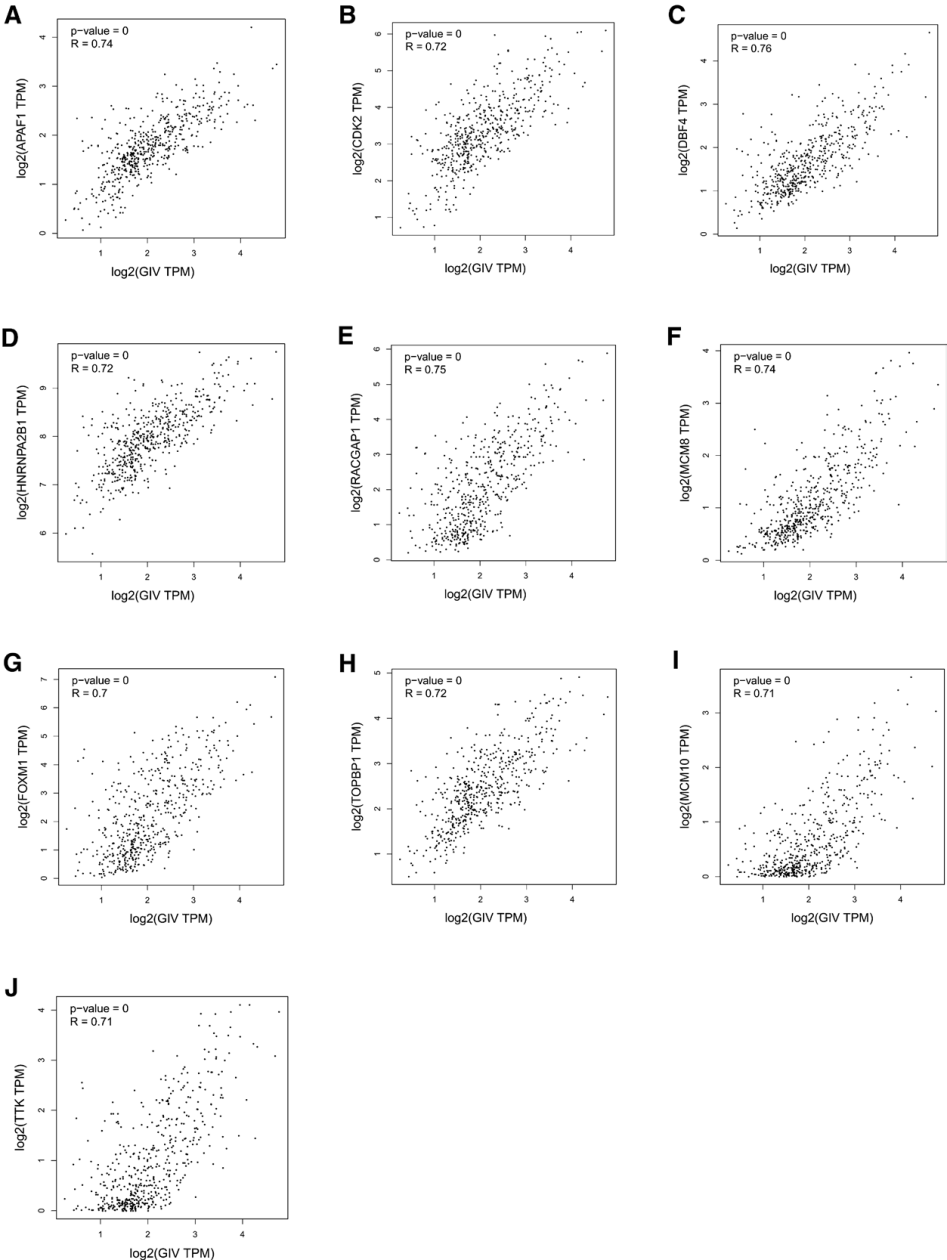
Correlation between the genes regulated by the key transcription factors and GIV. (A–J) The expression correlation between GIV and selected targeting genes. GIV = Gα-interacting, vesicle-associated protein, TPM = transcripts per million.

Using the TRUST tool, we assessed the prospective TF targets of the differentially expressed GIV-correlated genes and GIV-interacting genes. We found that the TFs E2F4 (FDR = 2.62 × 10^−5^), E2F1 (FDR = 1.51 × 10^−3^), MYC (FDR = 2.58 × 10^−2^), and MYCN (FDR = 3.05 × 10^−2^) are associated with GIV regulation (Table 2). GEPIA2 analysis of correlation between the genes regulated by the key TFs and GIV identified APAF1 (r = 0.74), CDK2 (r = 0.72), DBF4 (r = 0.76), HNRNPA2B1 (r = 0.72), MCM8 (r = 0.74), RACGAP1 (r = 0.75), TOPBP1 (r = 0.72), MCM10 (r = 0.71), TTK (r = 0.71), and FOXM1 (r = 0.70) as overlapping genes in patients with LIHC (Fig. 7A–J).

## 4. Discussion

Cancer is a major health concern, and its timely diagnosis and treatment are significant challenges. Globally, cancer mortality rates are significantly trending upward.^[20]^ Chinese liver cancer patients account for about 2% of the total global liver cancer burden and have a case fatality rate of 52%. Comprehensive studies of the molecular basis of tumorigenesis contribute to discovery of novel tumor markers as therapeutic targets.^[21]^ Mounting evidence shows that GIV influences tumor cell proliferation, migration, autophagy, and angiogenesis.^[22–24]^ GIV is highly expressed in the brain, kidney, heart, lung, spleen, testis, adipose tissue, and skeletal muscles.^[25,26]^ Some studies have identified a relationship between GIV, the TME, and tumor immunotherapy, illustrating that GIV regulates tumorigenesis and immunotherapy.^[27]^ Nevertheless, the prognostic significance along with the biological function of GIV in LIHC is unclear.

Here, we first assessed the abnormal expression of GIV through pan-cancer analysis and found the expression of GIV has also been confirmed in several tumors, which is consistent with previous reports.^[28]^ We also established that the expression of GIV in LIHC patients was higher than in nonmalignant tissues and that it is differentially expressed at various LIHC stages. Survival analysis of LIHC patients with GIV different expression revealed that the OS and DFS of 182 patients with high GIV expression were remarkably lower than that of those with low GIV levels, which is consistent with past findings.^[29]^ Analysis of differential GIV expression on cBioPortal showed that the GIV gene has a mutation rate of 1.3%. Next, the selected patients were grouped into the GIV mutation group and the nonmutation group for survival analysis. The Kaplan-Meier data illustrated that the median survival time of patients with variant GIV and nonvariant GIV was 8.61 and 83.57 months, respectively. In the disease-free group, the median survival time of patients with variant and nonvariant GIV was 2.73 and 29.69 months, respectively. In the OS group, the median survival time of patients with variant and nonvariant GIV was 4.6 and 60.89 months, respectively. In the absence of progress group, the median survival time of patients with variant and nonvariant GIV was 2.73 and 21.63 months, respectively. All differences were statistically significant. The results suggest that GIV expression changes in patients with LIHC can predict cancer development and prognosis.

The EPIC, MCPCOUNTER, XCELL, and TID analyses revealed that the expression level of GIV rose with the level of cancer-associated fibrosis. There is growing evidence that immune cell infiltration is crucial in immunotherapy and clinical response and that it affects tumor occurrence and development.^[30–33]^ CD4+ T cells recognize cancer antigens while activated M1 macrophages suppress tumor growth.^[34]^ Here, we found that the GIV expression positively correlated with invasion by B cells, dendritic cells, CD8+ T cells, macrophages, CD4+ T cells, and neutrophils, demonstrating that GIV has prognostic value and may reflect immune status. The major strength of this study was proved that GIV plays an important role in promoting cancer-inhibiting inflammation through the above algorithm.

Further, GO enrichment and KEGG analyses were used to study the function of GIV differential expression. We established the function of these genes is predominantly related to the cell cycle pathway. As an intracellular molecule, GIV can regulate cell growth and migration. Using TRUST, we found that E2F4, E2F1, MYC, and MYCN are the TF targets of GIV-related genes and GIV interaction genes in the TCGA database. GeneCardsSuite (human gene database, https://www.genecards.org/) revealed that E2F4 and E2F1 are of the E2F TF family. The E2F family regulates the cell cycle and tumor repressor proteins and is also a target for small DNA tumor virus transforming proteins. It mediates both cell proliferation along with p53-dependent/independent apoptosis. E2F1 preferentially binds to RB1 in a cell cycle–dependent approach and drives cell proliferation and TP53/p53-dependent apoptosis. E2F1 can promote the proliferation of LIHC cell line by activating B-Myb, stathmin 1, BRCA1, and DPB1. Studies have shown that E2F1 is significantly increased in LIHC tissues and is significantly correlated with tumor stages and poor prognosis of patients with LIHC.^[35]^ E1F4 is one of the repressors in the E1F family; microsatellite instability and E2F4 mutation are common in LIHC, indicating that they play an important role in LIHC.^[36]^ MYC is a multifunctional nuclear phosphoprotein and influences cell cycle progression, apoptosis, and cell transformation. It binds to the VEGFa promoter to promote VEGFa production, as well as subsequent budding angiogenesis. MYCN is an integral part of the MYC family, which encodes a protein with a basic helix-loop-helix domain. The protein resides in the nucleus and must bind to another basic helix-loop-helix factor in order to bind to DNA. MYCN amplification is implicated in numerous cancer types. Through the analysis of TFs, we found that GIV-correlated genes and GIV-interacting genes were involved in tumor angiogenesis and also participated in the occurrence and development of LIHC.

In conclusion, GIV upregulation is an adverse prognostic factor for patients with liver cancer and has therapeutic potential against liver cancer. It is expected to become a new tumor marker and therapeutic target. Further studies are required to validate our findings.

## Author contributions

All authors contributed toward data analysis, drafting, and critically revising the paper; gave final approval of the version to be published; and agree to be accountable for all aspects of the work.

## Supplementary Material


